# Body image resources for parents of youth: a scoping review

**DOI:** 10.3389/fpsyg.2026.1869014

**Published:** 2026-06-11

**Authors:** Madison F. Vani, Alishba Mansoor, Elise Christopoulos, Fengyue Xu, Landyn Meadows, Chelsi Ricketts, Catherine M. Sabiston

**Affiliations:** Faculty of Kinesiology and Physical Education, University of Toronto, Toronto, ON, Canada

**Keywords:** body image, parents, resource, scoping review, youth

## Abstract

**Introduction:**

Parents play an important role in shaping youth body image, yet many report uncertainty about how to provide supportive guidance, particularly in sport contexts where body image pressures are heightened. A scoping review was conducted to identify and synthesize existing parent resources to inform the development of an evidence-based, sport-specific parent resource for supporting youth body image.

**Methods:**

An eight-step scoping review methodology was used. Databases (Embase, PsycINFO, Social Work Abstracts, and MEDLINE accessed via Ovid, CINAHL, Gender Studies, and SportDiscus via EBSCO, and ERIC and Sociological Abstracts via ProQuest) were searched to identify relevant literature from January 1, 2014, to January 28, 2026. Four authors independently screened articles using predefined eligibility criteria and extracted data. Parents were consulted to gather feedback on the review findings. Data were analyzed using descriptive statistics and qualitative content analysis.

**Results:**

Fifteen articles met inclusion criteria (9 quantitative, 3 qualitative, 2 mixed methods, 1 review), representing ten unique resources. Most resources were programs or guidelines delivered in online text or workshop/session formats. Resources commonly discussed body image influences, positive body image, and health behaviours. Few resources were tailored to adolescents or diverse identity groups, and most encouraged parents to provide youth informational and emotional support. Findings from the parent consultation demonstrated a preference for practical, accessible, and inclusive resources.

**Discussion:**

Overall, results highlight the need for evidence-informed, developmentally appropriate, and culturally responsive parent resources to support youth body image in sport. Future efforts should prioritize co-design, evaluation, and integrated dissemination to maximize impact.

**Systematic review registration:**

https://osf.io/9hq78, identifier.

## 1 Introduction

Body image is a multidimensional construct encompassing individuals’ perceptions, thoughts, feelings, and behaviours related to their body’s appearance and function (Cash and Smolak, 2011). According to sociocultural perspectives of body image (Thompson et al., 1999; Tiggemann, 2011), sources that influence the development and maintenance of body image include parents, peers, and the media. Within Western cultural contexts, these influences often convey dominant appearance ideals that prioritize thinness for girls and muscularity for boys (Tiggemann, 2011). The sources elicit a greater focus on appearance and function through behaviours such as body commentary, modeling appearance concerns, and reinforcing idealized appearance and functionality expectations, which can increase body image concerns among youth (Hart et al., 2015; Tiggemann, 2011). Such concerns have been documented in children as young as six years and tend to intensify in adolescence (Markey, 2010; Lowes and Tiggemann, 2003; Voelker et al., 2015). Body image concerns among youth are associated with poorer psychological and physical well-being, including increased depressive symptoms and disordered eating (Ferreiro et al., 2014; Paxton et al., 2006). Given the early onset and detrimental impacts associated with body image concerns, identifying effective strategies to prevent and reduce these concerns is critically important. As primary sources of socialization, parents, guardians, and caregivers (here forth termed ‘parents’) are uniquely positioned to support their children’s body image and overall well-being, highlighting the importance of identifying resources available to support parents in this role.

Parents’ communication and behaviours contribute to shaping children’s developing body image. Specifically, parents can model body image concerns through dieting, body checking, and negative body comments directed at the self (Tremblay and Limbos, 2009). In fact, perceptions of mothers’ body dissatisfaction predicted 5–8-year-old girls’ and boys’ body dissatisfaction (Lowes and Tiggemann, 2003). Further, parents’ direct body and weight commentary and rules or expectations around eating, exercise, and weight also contribute to their children’s body image concerns (Rodgers and Chabrol, 2009; Tremblay and Limbos, 2009). Conversely, parents displaying self-compassion, modeling healthy behaviours (e.g., intuitive eating), avoiding body comparisons, vocalizing their unconditional acceptance of their child’s body, and normalizing open communication can foster more positive body image (Goslin and Koons-Beauchamp, 2023; Rodgers et al., 2024). These behaviours can be understood through the lens of social support, which includes emotional, informational, tangible, esteem, and validation support, and provides a useful framework for categorizing the strategies parents use (Rees and Hardy, 2000; Wills and Shinar, 2000). Using this framework can help organize parental recommendations and inform the development of resources that target specific types of social support. Despite the established parental influence on youth body image, many parents report limited confidence about how to effectively model and engage in supportive communication around body image, particularly as children navigate varying social environments (Koulanova et al., 2021; Rodgers et al., 2024; Siegel et al., 2021).

Parents’ influence on youth body image is also shaped by the contexts their children navigate, including sport environments that present unique pressures related to body image. Sport environments heighten attention on appearance and weight, are evaluative, require uniforms that may be uncomfortable or revealing, motivate body comparisons, and create opportunities for commentary on appearance and weight (Koulanova et al., 2021; Lucibello et al., 2025; Matheson et al., 2023; Vani et al., 2021). Importantly, these pressures are situated within broader Western societal ideals that prioritize thinness and muscularity (Lunde and Gattario, 2017). In fact, youth in sport often navigate both sport-specific body ideals and broader societal body ideals, which can conflict and create additional tension (Lunde and Gattario, 2017). Consistent with these pressures, youth in sport commonly report experiencing body image concerns (Koulanova et al., 2021; Matheson et al., 2023; Sabiston et al., 2019), which contribute to lower sport enjoyment and commitment (Pila et al., 2020) and higher rates of sport disengagement (Vani et al., 2021). Within this context, parents play a key role in promoting safety and support for their child’s well-being, as they may buffer or inadvertently reinforce sport-specific body-related pressures. They also need to identify and respond to the competing body-ideal pressures their children face. However, many parents report feeling uncertain about how to recognize or address body image concerns, and youth may not always feel comfortable discussing concerns with their parents (Koulanova et al., 2021). Strengthening parents’ capacity to provide informed and supportive body image guidance is therefore critical, especially given the well-established benefits of sport participation (Eime et al., 2013; Doré et al., 2019) and the harmful consequences of body image concerns (Ferreiro et al., 2014; Paxton et al., 2006). Yet, to our knowledge, no dedicated resources exist to help parents navigate body image concerns within sport settings.

Only one review has summarized the resources available to parents to support youth body image. This review focused solely on intervention studies and on a specific aspect of body image (i.e., body dissatisfaction; Hart et al., 2015), which limits understanding of the broader study of body image and excludes diverse resource formats (e.g., recommendations). In addition, the article was designed as a systematic review with a narrower methodological scope (Hart et al., 2015). In contrast, a scoping review is a form of knowledge synthesis well suited to exploratory objectives and offers a broad approach to identifying articles that have developed resources for parents in various formats (Sabiston et al., 2022). To begin the process of developing a parent-focused body image resource for sport, it is necessary to first synthesize the broader scope of existing resources across any context.

The purpose of this scoping review was to examine the available resources for parents on youth body image. Three objectives guided the review: (1) identify how many resources exist for parents to support youth body image; (2) illustrate the resources’ characteristics (i.e., resource type/format; method(s) used to develop resource); and (3) describe the topics/content covered in the resource. The findings will provide a comprehensive synthesis of evidence-based guidance by identifying common resource types, formats, and content, including key topics and recommendations, found in general body image resources for parents. These results will further inform the co-design of a parent resource aimed at supporting youth body image in sport.

## 2 Materials and methods

The current scoping review process followed an established eight-step methodological framework that was applied using an iterative approach (Sabiston et al., 2022). Reporting of this scoping review aligns with the Preferred Reporting Items for Systematic Reviews and Meta-Analyses – Extension for Scoping Reviews (see Supplementary file 1 for checklist; PRISMA-ScR; Tricco et al., 2018).

### 2.1 Step 1: create and consult a knowledge user group

Aligned with the guidelines established by Sabiston et al. (2022), knowledge users are typically engagend at two points: (a) an initial consultation to gather feedback on the scoping review methods, and (b) a reaction meeting to provide input on the interpretation of findings, reporting of results, and strategies for knowledge dissemination (see Step 8 for further details). However, due to practical constraints, initial consultations with parents were not conducted. The foundation for this scoping review was informed by prior conversations with sport leaders (e.g., organizations, coaches, parents) and insights from previously conducted interviews with parents of youth in sport (Koulanova et al., 2021). These early discussions played a key role in shaping the development of the methods. Additionally, as described in later steps, the research team sought and incorporated feedback on the methods from topic experts and a health sciences librarian.

### 2.2 Step 2: identify the research questions

The scoping review was guided by three research questions: (1) How many resources exist for parents to support youth body image?; (2) What are the characteristics (i.e., resource type/format; method(s) used to develop resource) of the resources for parents to support youth body image?; and (3) What are the topics and content covered in the resources for parents to support youth body image? The PCC mnemonic (Peters et al., 2020) was used to develop the research questions to explore the population of parents, concept of body image, and context of parent-focused resources. The components were defined during protocol development and are outlined below.

#### 2.2.1 Population

Parents, caregivers, and guardians of youth aged 4–18 years (referred to as ‘parents’ for brevity) comprised the population. In this review, the term ‘youth’ was used to refer to children and adolescents. The lower age range of 4 years was used to include preventative resources, as body image concerns have been reported as early as age six (Lowes and Tiggemann, 2003). An upper limit of 18 years was selected to align with commonly used psychological definitions of adolescence (American Psychological Association, 2002). Resources created for parents and focused on youth were eligible, inclusive of all identity factors (e.g., gender identity, weight status).

#### 2.2.2 Concept

The concept of body image was defined as a multidimensional construct that involves perceptions, cognitions, affect, and behaviours related to the body’s appearance and function (Cash and Smolak, 2011). In line with this broad definition, any resources addressing one or more components of body image (e.g., body commentary, body appreciation, body dissatisfaction) were eligible.

#### 2.2.3 Context

The context included scholarly materials developed for parents that offered guidance or recommendations related to youth body image. Various synonyms of resources (e.g., guides, recommendations, guidelines, toolkits, frameworks) were eligible.

### 2.3 Step 3: identify relevant studies

#### 2.3.1 Information sources

Nine electronic databases were searched: Embase, PsycINFO, Social Work Abstracts, and MEDLINE accessed via Ovid, CINAHL, Gender Studies, and SportDiscus via EBSCO, and ERIC and Sociological Abstracts via ProQuest. In addition, reference lists of included sources were manually searched.

#### 2.3.2 Search strategy

Initial searches were conducted in two databases to explore terms related to the population and concept. These exploratory searches helped identify relevant keywords and index terms. The final strategy incorporated controlled vocabulary (e.g., MeSH in MEDLINE and PsycINFO, EMTREE in Embase, CINAHL Headings in CINAHL) and free-text terms, using Boolean logic and operators. The search strategy was refined with input from a health sciences librarian and five topic experts in body image and/or review methodology, who were identified through the research team’s networks. These experts peer-reviewed the complete search strategy using the CADTH Peer Review Checklist for Search Strategies (McGowan et al., 2016) to enhance rigor and ensure accurate translation across databases. The final search was conducted on February 11, 2025. An updated search was conducted on January 28, 2026 to identify any articles published since the final search. The full search strategy is presented in Supplementary file 2.

#### 2.3.3 Inclusion/exclusion criteria

Sources were included if they met the following criteria: (a) qualitative, quantitative, mixed methods, or review designs (e.g., systematic reviews, scoping reviews, meta-analyses); (b) written in English; (c) published between January 1, 2014, and January 28, 2026; (d) included a resource (e.g., guidelines, recommendations); (e) the resources were designed for parents of youth aged 4–18 years (without needing to cover the entire age range); and (f) the resources addressed at least one aspect of body image (e.g., body/weight commentary, body appreciation, body confidence). The search dates were limited to capture more recent resources that reflect current conceptualizations of body image (Cash and Smolak, 2011; Tylka and Wood-Barcalow, 2015) and offer information most relevant for informing the development of a new sport-specific resource. Sources were excluded if they: (a) were editorials, preprints, protocols, theses, dissertations, conference or meeting abstracts and proceedings, commentaries, and book chapters; (b) discussed suggestions for parents to support youth body image without explicitly including developed resources; (c) focused on related constructs (e.g., self-esteem) to body image without an inclusion of body image concepts; and (d) clinical or treatment-seeking populations (e.g., individuals with cancer, clinically diagnosed mental illness, or those undergoing medical weight management).

### 2.4 Step 4: create and register a protocol

A protocol was pre-registered with Open Science Framework on February 11, 2025^1^ and is published (Vani et al., 2026). The protocol is reported in accordance with the evidence-based guidelines (Sabiston et al., 2022) and PRISMA-P checklist (Moher et al., 2015).

### 2.5 Step 5: select and screen studies

The search was conducted by the first author (MFV), and the results were imported into Covidence (Covidence software: Covidence systematic review software, 2015), where duplicate records were identified and removed. Study selection was conducted in two stages using pre-established eligibility criteria: (1) title and abstract screening, and (2) full-text review. Four independent reviewers (AM, EC, LM, FX) screened articles in duplicate. Disagreements between reviewers were resolved through discussion; if consensus could not be reached, a fifth reviewer (MFV) made the final decision. To update the search, MFV conducted the search and two independent reviewers (MFV, AM) screened articles in duplicate. Disagreements were resolved through discussion.

### 2.6 Step 6: chart the data

A chart form was used to extract and organize the results into a descriptive summary. As a pilot exercise for consistency, AM, EC, LM, and FX independently extracted data in duplicate from the first five sources. Following the pilot, reviewers met to discuss challenges and consulted MFV for a final decision on discrepancies. Data extraction for the remaining sources was carried out in duplicate by the same four reviewers, with any conflicts resolved by MFV. The charting process followed an iterative approach, allowing for modifications to the form throughout extraction (Sabiston et al., 2022). The final extracted data included: author, year, country, study design, study purpose, type of resource, format of resource, method for developing resource, resource topic(s), recommendations, identity factors, evaluation design, population and sample size, evaluation measures, and main findings.

### 2.7 Step 7: collate, summarize, and report the results

Data were analyzed using a descriptive numerical summary and qualitative content analysis (Hsieh and Shannon, 2005). Frequencies were calculated to provide an overview of the included sources and results. One team member (MFV) conducted the content analysis by reading and re-reading the included studies, generating initial codes with reference to the data charting form, and organizing these codes into categories based on their relationships. The broader review team contributed to the interpretation of findings, with remaining members serving as critical friends to offer alternative perspectives and enhance analytical rigor (Smith and McGannon, 2018). The analyses and presentation of results were guided by the research questions, focusing on the: (1) number of existing resources, (2) resource characteristics, and (3) body image topics/content addressed. When analyzing resource content, a content analysis with a directed approach (Hsieh and Shannon, 2005) was further used to sort resource recommendations into social support categories (i.e., tangible, emotional, esteem, informational, validation; Rees and Hardy, 2000; Wills and Shinar, 2000). In addition, findings were summarized based on characteristics related to the study, population, concept, context, and any reported outcomes. Results were also informed by the PRISMA-ScR Checklist (e.g., presentation of PRISMA flow diagram) and feedback from knowledge users. Findings are presented descriptively, thematically, and in summary tables (Tricco et al., 2018).

### 2.8 Step 8: consult knowledge users and consider implications

Knowledge-user engagement processes were approved by the University of Toronto research ethics board (protocol number: 00047474; date of approval: May 12, 2025). Parents were recruited through social media, previous participant lists (with consent for future research), and snowball sampling. To be eligible, parents had to meet the following criteria: (a) be 18 years or older; (b) have a child aged 4–18 years who participates in sport at any level; and (c) be proficient in reading, speaking, and understanding English. Informed consent was obtained from all participants prior to completing a brief demographic and scheduling survey and participating in the focus group.

To enhance the applicability of the scoping review findings (Sabiston et al., 2022), parents who met eligibility criteria were invited to participate in a reaction meeting. One focus group was conducted. Parents (*n* = 7) were on average 43.00 ± 4.04 years of age (range = 36–49), identified as women (100%), and most reported current engagement in sport or physical activity/recreation (71.4%). They reported their highest level of education as undergraduate degree (28.6%), post-graduate diploma or certificate (14.3%), professional program (14.3%), master’s degree (28.6%), or doctorate degree (14.3%). Parents identified as White (57.1%), South Asian (14.3%), mixed race (14.3%), or prefer not to answer (14.3%). Finally, family’s average subjective socioeconomic status was 7.33 ± 1.21 (*n* = 6 parents responded to this question; score range = 5–8; scale: 0 = people with the least money, education, respected jobs to 10 = people with the most money, education, respected jobs).

The focus group lasted 90 min and was conducted via Zoom by three team members (co-facilitators: MFV and CR; note-taker: AM). A semi-structured focus group guide was used to facilitate discussion, with the aim of gathering participants’ perspectives on the scoping review results and exploring implications, particularly in relation to the development of a parent-focused resource for supporting youth body image in sport. During the focus group, knowledge users were invited to share ideas on potential dissemination strategies (e.g., social media content) to help ensure the findings reach diverse parent and expert audiences. Additionally, in consultation with parents, future directions for research and practice were discussed.

To analyze focus group data, audio recordings were transcribed verbatim and a qualitative content analysis was used (Hsieh and Shannon, 2005). MFV conducted the analysis by reading the transcript, generating codes, and creating categories based on relationships between the codes. Findings were then shared and discussed with the broader research team, with remaining members serving as critical friends to provide constructive feedback and challenge interpretations (Smith and McGannon, 2018). Insights from focus group discussions were integrated into the results and discussion.

## 3 Results

**Figure 1:**
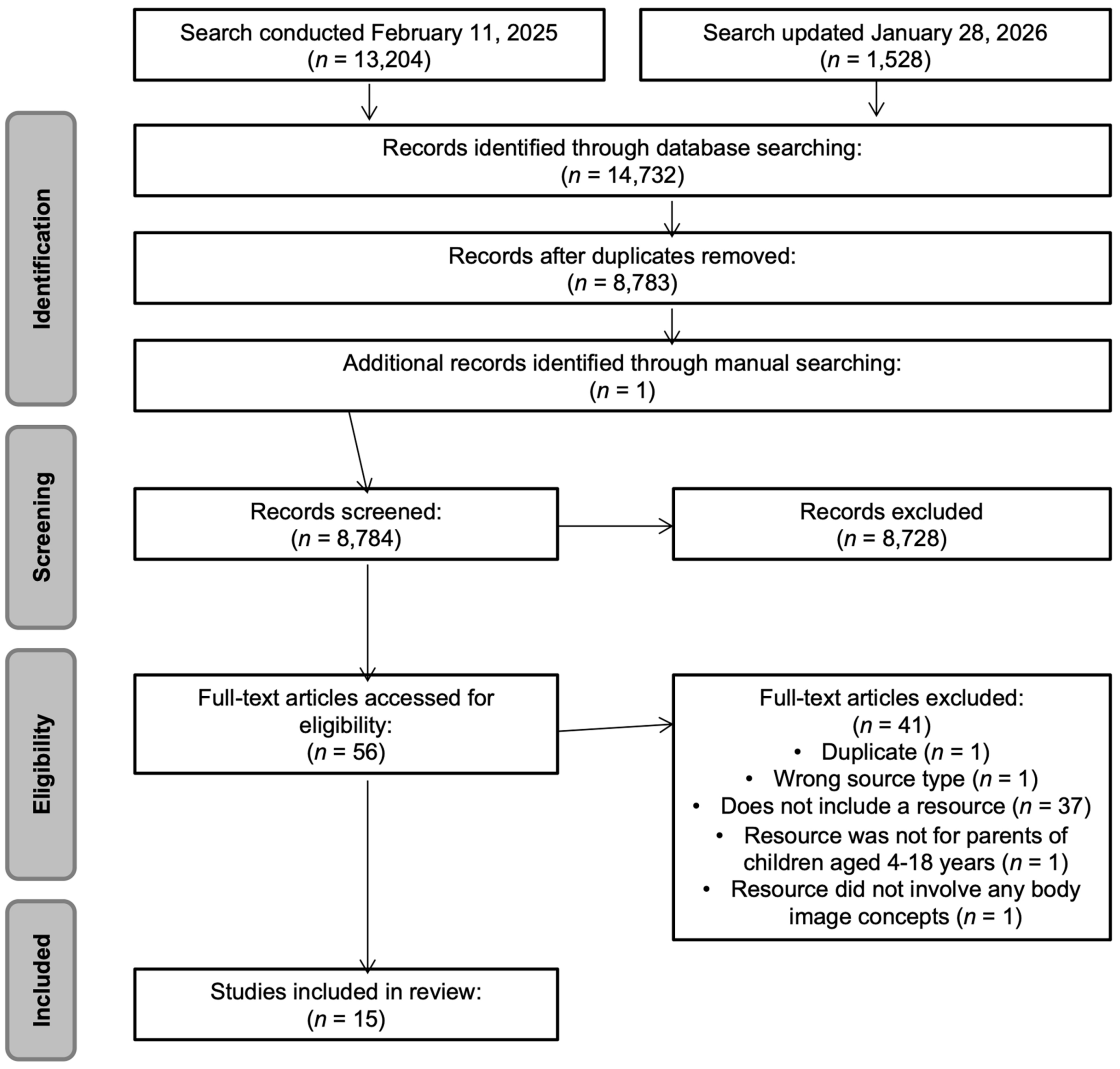
PRISMA flow diagram.

The database search identified 14,732 records. After removing duplicates and adding one article identified through manual searching, 8,784 title and abstracts were reviewed for eligibility. Following the first stage of screening, 56 full texts were screened. Fifteen articles met inclusion criteria and were included in this review (see Figure 1 for PRISMA flow diagram).

### 3.1 Study characteristics

The articles were published between 2014 and 2025 from a range of geographical locations including Australia (*n* = 8), United States of America (*n* = 4), England (*n* = 4), Canada (*n* = 2), Ireland (*n* = 1), and New Zealand (*n* = 1). Note Carbonneau et al. (2021), Diedrichs et al. (2016), Meskin et al. (2021), and Twomey and Gillison (2025) were multi-country collaborations. Study designs included quantitative (*n* = 9), qualitative (*n* = 3), mixed methods (*n* = 2), and review (*n* = 1). Additional study characteristics are presented in Supplementary file 3, Supplementary Table S1.

### 3.2 Resource characteristics

Three articles reported on the same guidelines (Baber et al., 2023; Gillison et al., 2023; Twomey and Gillison, 2025) and five articles reported on the development (Hart et al., 2014) or evaluation (Hart et al., 2016, 2019; Hill et al., 2020; Meskin et al., 2021) of the same overarching resource. Given Hart et al. (2014) described an earlier version of the resource, it was analyzed separately. Therefore, 10 unique resources are described. Resources were classified as programs (*n* = 4; Eldridge et al., 2016; Forbes et al., 2024; Hart et al., 2016, 2019; Hill et al., 2020; McCabe et al., 2016; Meskin et al., 2021), guidelines (*n* = 2; Baber et al., 2023; Gillison et al., 2023; Hart et al., 2014; Twomey and Gillison, 2025), recommendations (*n* = 1; Carbonneau et al., 2021), a co-created theory (*n* = 1; Horton, 2024), an educational video (*n* = 1; Klupt et al., 2020), and a website information hub (*n* = 1; Diedrichs et al., 2016). Among the 10 unique resources, they were mainly delivered in an online text format (*n* = 6), followed by workshops/sessions (*n* = 4), print (*n* = 3), and online video format (*n* = 3). See Supplementary file 3, Supplementary Table S1 for each article’s resource characteristics.

Resources were developed for parents of children and/or adolescents, with 13 of the articles reporting age information of youth ranging from 2 to 18 years. In fact, some resources (*n* = 8) were designed for specific age groups, including parents of children under 12 years (*n* = 5; Baber et al., 2023; Eldridge et al., 2016; Gillison et al., 2023; Hart et al., 2014, 2016, 2019; Hill et al., 2020; McCabe et al., 2016; Meskin et al., 2021; Twomey and Gillison, 2025), adolescents (12–18 years, *n* = 1; Forbes et al., 2024), or both children and adolescents (*n* = 2; Diedrichs et al., 2016; Horton, 2024). In addition, three resources were specifically created for parents of boys (McCabe et al., 2016) or girls (Diedrichs et al., 2016; Forbes et al., 2024).

In consideration of context, one program was designed for delivery within schools (Forbes et al., 2024), while another was created specifically for parents residing in rural communities (Eldridge et al., 2016). In terms of culture, within the articles, eight authors (Baber et al., 2023; Gillison et al., 2023; Hart et al., 2016, 2019; Hill et al., 2020; Horton, 2024; Klupt et al., 2020; Meskin et al., 2021) acknowledged cultural considerations. In particular, seven articles mentioned the lack of cultural diversity in the parent samples or the need for testing with a diverse group (Baber et al., 2023; Gillison et al., 2023; Hart et al., 2016, 2019; Hill et al., 2020; Horton, 2024; Klupt et al., 2020), while two articles discussed the need for a cultural adaptation of the resource (Baber et al., 2023; Meskin et al., 2021).

### 3.3 Resource development

Thirteen articles described how the resource was developed (Baber et al., 2023; Carbonneau et al., 2021; Eldridge et al., 2016; Forbes et al., 2024; Gillison et al., 2023; Hart et al., 2014, 2016, 2019; Hill et al., 2020; Horton, 2024; Klupt et al., 2020; Meskin et al., 2021; Twomey and Gillison, 2025). Most authors used multiple methods to create their resource, including a literature, scoping, or systematic review or systematic search (*n* = 12), interviews, focus groups, or a survey with parents (*n* = 9), Delphi study (*n* = 8), pilot/feasibility/acceptability study (*n* = 5), interviews or a survey with experts (*n* = 4), and interviews with children (*n* = 1). Of the 13 articles that discussed resource development, the purpose of three articles was specifically to develop the resource (Gillison et al., 2023; Hart et al., 2014; Horton, 2024). Authors described using interviews with children and a Delphi process with parents and experts (Gillison et al., 2023), a Delphi method with experts (Hart et al., 2014), and interviews with parents (Horton, 2024). See Supplementary file 3, Supplementary Table S1 for individual study details.

### 3.4 Resource content

Only three resources primarily focused on body image (Forbes et al., 2024; Horton, 2024; McCabe et al., 2016). The remaining centered weight or obesity (Baber et al., 2023; Eldridge et al., 2016; Gillison et al., 2023; Klupt et al., 2020; Twomey and Gillison, 2025), food/healthy eating practices (Carbonneau et al., 2021; Hart et al., 2014, 2016, 2019; Hill et al., 2020; Meskin et al., 2021), or self-esteem (Diedrichs et al., 2016) either as a focus or alongside body image. Resources involved a range of body image topics (see Table 1). All resources considered body image influences (*n* = 10), such as body commentary (*n* = 9) and guidance on effective communication about body image (*n* = 9). Positive body image concepts were embedded into all resources (*n* = 10); common facets included body acceptance, body and/or functionality appreciation, and body respect. In contrast, negative body image appeared less frequently (*n* = 6), while body neutrality was addressed in one resource (Horton, 2024). Discussions of identity and demographic variables in relation to body image were commonly centered around gender (*n* = 7), weight (*n* = 5), and age (*n* = 4). One resource explicitly stated that the resource may not be suitable for cultural groups outside of English-speaking countries (Hart et al., 2014). Finally, most resources incorporated messaging about health behaviours, with food/nutrition (*n* = 9) and physical activity (*n* = 9) being the most prevalent. However, despite discussion of physical activity as a health behaviour, none of the identified resources were specifically tailored to sport or physical activity contexts.

**Table 1:** Body image topics included in resources (*N* = 10).

Category	Description	*n* (%)
Positive body image	The resource discussed facets of positive body image	10 (100%)
	Body acceptance	7 (70%)
	Body/functionality appreciation	6 (60%)
	Body respect	6 (60%)
	Body confidence	5 (50%)
	Broad conceptualization of beauty	3 (30%)
	Body pride	1 (10%)
Negative body image	The resource discussed facets of negative body image	6 (60%)
	Body dissatisfaction	3 (30%)
	Body shame	2 (20%)
	Body self-consciousness	1 (10%)
Body neutrality	The resource discussed body neutrality	1 (10%)
Body image influences	The resource discussed factors influencing body image	10 (100%)
	Body talk/commentary	9 (90%)
	Parent–child communication	9 (90%)
	Parental influence on body image	8 (80%)
	Weight talk/commentary	7 (70%)
	Media influence on body image	7 (70%)
	Body-related teasing/bullying	6 (60%)
	Internalization of body ideals	5 (50%)
	Body/appearance comparisons	3 (30%)
	Peer influence on body image	4 (30%)
	Weight stigma	1 (10%)
Identity/demographics	The resource discussed the intersection of body image with identity or demographic factors	9 (90%)
	Gender	7 (70%)
	Weight	5 (50%)
	Age	4 (40%)
	Puberty	1 (10%)
	Culture	1 (10%)
Health behaviours	The resource discussed health behaviours and their relationship with body image	9 (90%)
	Physical activity/movement	9 (90%)
	Food/nutrition/eating	9 (90%)
	Sleep	2 (20%)

Results are displayed as absolute frequencies, as resources can be classified into multiple categories and subcategories.

Within most of the resources (*n* = 7), parents were mainly encouraged to take both proactive (i.e., prevention of problems before they arise; e.g., modeling positive body image behaviours) and reactive (i.e., responding to situations as they happen; e.g., listen and offer support when child expresses body image concerns) roles. The remaining three resources recommended proactive approaches only (Carbonneau et al., 2021; Horton, 2024; McCabe et al., 2016). Resources included recommendations that were interpreted as informational support (*n* = 10), followed by emotional (*n* = 9), esteem (*n* = 7), tangible (*n* = 6), and validation (*n* = 5) support. See Table 2 for example recommendations within the social support types.

**Table 2:** Social support types included in resource recommendations (*N* = 10).

Type	Description	*n* (%)	Examples
Informational	The resource recommended parents provide child with information about resources, suggest alternative courses of action, or offer advice	10 (100%)	Model positive body image behaviours and attitudes (McCabe et al., 2016) Model body neutral language (Horton, 2024)
Emotional	The resource recommended parents allow child to discuss feelings, express concerns/worries, and show sympathy, approval, caring, and acceptance of their child	9 (90%)	Listen and offer support when child expresses weight-related concerns (Klupt et al., 2020) Display acceptance and non-judgemental stance toward child’s appearance (Carbonneau et al., 2021)
Esteem	The resource recommended parents bolster/support child’s confidence or self-worth, encourage child	7 (70%)	Focus on child’s strengths and uniqueness (Forbes et al., 2024) Praise child for a variety of things beyond appearance (Baber et al., 2023; Gillison et al., 2023)
Tangible	The resource recommended parents provide child with money, household goods, tools, transportation, and/or assistance with cooking/shopping/ cleaning	6 (60%)	Seek professional assistance if you suspect child has body image concerns (Hart et al., 2016, 2019; Hill et al., 2020; Meskin et al., 2021) Create an accessible physical environment that promotes an active lifestyle (Eldridge et al., 2016)
Validation	The resource recommended parents to provide consensus information regarding prevalence of problems to their child and normalize child’s behaviour and feelings	5 (50%)	Normalize puberty-related body changes (Hart et al., 2014) Validate that it is normal to feel embarrassed in certain settings and friends likely feel similar (Baber et al., 2023; Gillison et al., 2023)

Support type descriptions were informed by social support conceptualizations (Rees and Hardy, 2000; Wills and Shinar, 2000).

### 3.5 Resource testing/evaluation

Eleven articles described an evaluation of the resource (Baber et al., 2023; Diedrichs et al., 2016; Eldridge et al., 2016; Forbes et al., 2024; Hart et al., 2016, 2019; Hill et al., 2020; Klupt et al., 2020; McCabe et al., 2016; Meskin et al., 2021; Twomey and Gillison, 2025). Evaluations involved interviews with parents and experts (Baber et al., 2023; Twomey and Gillison, 2025), play-based interviews with children whose parents participated in a randomized controlled trial (Hill et al., 2020), single arm pilot trials assessing resource acceptability and effectiveness (Klupt et al., 2020; Meskin et al., 2021), quasi-experimental designs assessing program effectiveness (Eldridge et al., 2016) or acceptability and efficacy (Forbes et al., 2024), and randomized controlled trials assessing resource effectiveness (Diedrichs et al., 2016; Hart et al., 2016, 2019; McCabe et al., 2016).

In seven articles, only parents were assessed, with outcomes measured across cognitive (e.g., parents’ own body appreciation; Forbes et al., 2024; McCabe et al., 2016; Meskin et al., 2021) and behavioural (e.g., parents’ own weight control behaviours and parent modeling, Forbes et al., 2024) dimensions of body image. Additional body image-related outcomes were commonly included, such as perceived knowledge related to body image (Hart et al., 2016, 2019; McCabe et al., 2016; Meskin et al., 2021). Finally, resource feedback (e.g., acceptability, feasibility) was measured in three articles (Baber et al., 2023; Forbes et al., 2024; Klupt et al., 2020). Two articles only included youth and assessed perceptual (*n* = 1; e.g., weight and body size perception, Eldridge et al., 2016), cognitive (*n* = 2; e.g., body satisfaction; Hill et al., 2020; weight satisfaction, Eldridge et al., 2016), and behavioural (*n* = 2; e.g., weight control behaviours, Eldridge et al., 2016; unhealthy eating pattern, Hill et al., 2020) body image outcomes. One article (Diedrichs et al., 2016) included parent and youth assessments, measuring cognitive (e.g., appearance and weight esteem) and behavioural (e.g., appearance-based conversations) body image, body image influences (e.g., internalization of appearance ideals), psychosocial outcomes (e.g., negative affect), and mothers’ adherence to resource engagement. Finally, acceptability and usability were assessed with health care practitioners in one article (Twomey and Gillison, 2025). Additional details (i.e., measures, results) are in Supplementary file 3, Supplementary Table S2.

### 3.6 Consultation with parents

Seven parents participated in a focus group to provide their perspectives on the review results. Two themes were generated that demonstrate the parents’ perceptions of the existing resources and ideas for future resource development, as well as knowledge dissemination.

#### 3.6.1 Perceptions of existing resources and priorities for future resource development

Within five subthemes, parents reflected on the scoping review results by identifying limitations of the existing resources and key priorities for future resource development to improve relevance and inclusivity.

##### 3.6.1.1 Developmentally appropriate resources

Most parents were surprised that resources focused on children under 12 years of age. While parents saw value in instilling “body confidence early because then when they are teens they have…confidence,” they also felt that resources designed for adolescents are needed. One parent shared, “…knowing that when kids are going through puberty, and when they are sort of experiencing the most anxiety around their bodies changing […] I thought I would see more focus in that area.” Further, parents agreed that body image resources designed for age cohorts would be valuable. For example, in the context of sport, a parent recommended aligning resources with long-term athlete development milestones, suggesting that body image topics could be paired with youth’s physical, cognitive, and emotional development. Another parent underscored the importance of considering development when they stated that “if a child…has hit puberty before others it can be difficult.” Taken together, these perspectives highlight a need for developmentally attuned body image resources that address the unique challenges youth experience over time.

##### 3.6.1.2 Cultural and racial identity relevance

Parents also emphasized the need for resources that meaningfully engage with cultural diversity, an identity factor they noted was markedly missing from existing resources. Specifically, they expressed a desire for guidance on navigating conversations about race and cultural identity with their children, including topics related to hair texture and skin colour. For instance, one parent shared about their nephew who identifies as mixed race, “there were lots of comments made, just based on his skin colour.” They went on to suggest that parents need support to navigate:

how to talk to kids […] biracial kids…the parent that’s maybe comfortable having these conversations is the one that’s not of the same race...there’s a lot of education to do for parents who don’t understand what their kid may be experiencing.

Overall, parents highlighted the importance of culturally responsive and inclusive body image resources that equip them to support children’s identity experiences with confidence and sensitivity.

##### 3.6.1.3 Moving beyond weight: supporting diverse body experiences

In addition to developmental and cultural considerations, parents emphasized that children’s body image concerns extend beyond weight, yet many existing resources conflated the two. They described a need for resources that acknowledge and support a wide range of body-related experiences, including height, hair texture, strength, and ability. For example, one parent shared that their child, “…gets overlooked because of her height […] It’s not really the weight part for me. There’s other things that are still related to body image or culture that have had an impact on how she feels.” Some parents also noted that using strengths-based language (e.g., emphasizing strength or speed) can help to redirect attention away from body concerns, including those related to weight.

##### 3.6.1.4 Proactive and reactive strategies for parents

Parents acknowledged the need for both proactive and reactive approaches for supporting their children, however, many parents emphasized the particular importance of proactive strategies. Parents identified a need for resources that help them identify early signs of body image concerns, recognize subtle shifts in behaviour, and initiate open-ended conversations without leading or pathologizing the issue. For example, one parent of a seven-year-old shared, “so for me, it’s about those proactive resources and looking out for what signs, what language is being used, what behaviour changes could be there, if she’s not so forthcoming in what’s being said.” In contrast, one parent expressed a clear desire for a combination of proactive and reactive support strategies related to body commentary. In addition to example prompts for checking in with children and creating a safe space that fosters sharing, the parent also described a need for evidence-informed guidance to help their child unpack body commentary they receive. Specifically, they shared a desire for “how to work through those [body] comments that children can get hung up on […] we are the ones that are their sounding boards when they have those feelings and then need support going through them.” Together, these reflections illustrate the value parents place on preventative guidance, while still recognizing the need for responsive support when challenges arise.

##### 3.6.1.5 Additional needs: language, format, delivery

Practical needs related to the design and delivery of the resources were identified by parents. Overarchingly, parents highlighted the importance of considering strengths-based, trauma-informed language when developing resources. When discussing resource type, parents expressed a clear preference for recommendations, followed by educational videos. Meanwhile, guidelines and programs were not favourable to most parents. In terms of delivery format, online text resources and virtual workshops or sessions were strongly preferred, while none of the parents desired in-person sessions. Some parents were open to print or online video options. Finally, parents felt that the resources depend on alignment with the broader adult environment surrounding youth. Several parents noted that inconsistent or harmful body-related messaging from coaches, teachers, or early childhood educators can undermine parents’ efforts. For example, one parent shared, “you can only do so much at home, but if the coach is the one that’s giving negative comments, it feels a bit backwards […] more than just parents, also coaches need to be educated on positive ways of framing.” This underscores the need for coordinated approaches to body image support for youth.

#### 3.6.2 Barriers and preferences for access and dissemination

Parents felt that the results of this scoping review should be shared with parents, other family members (e.g., grandparents), school leaders (e.g., teachers, administration), and sport leaders (e.g., coaches, team managers) through social media, infographics, websites, and/or workshops or training sessions. However, the parents were unaware of any parent resources to support youth body image. They expressed that if they needed help to support their child’s body image, they would turn to a trusted source or a mental health professional (e.g., psychologist, counselor, sport association). For example, one parent shared, “I want something directly from a trusted resource in the field that I’m looking in… I’d want the professionals to be giving me the specific resource.” Building on this sentiment, a parent outlined the difficulty of engaging parents who may especially benefit from accessing the resources:

…you probably have a population of parents here who have talked about these things at home […] but I do know that there’s parents that aren’t. […] it needs to come through the organizations or coaches or trusted allies to get it to the right people.

In addition to the source sharing the resource, parents spoke about the importance of the format of receiving information. Parents lack time for in-person delivery and are inundated with emails, however, they did express that meeting parents where they already are would be most effective for knowledge and resource dissemination. One parent shared that their child’s sport association “requires parents to complete an online module prior to your child being allowed to take part in any [sport association] competitions. So, it would be helpful to include those resources in these mandatory courses.” Collectively, parents recognized the value of the resources but stressed that meaningful access and effective dissemination required trusted sources, convenient formats, and integration into existing parent obligations.

### 3.7 Integrated findings

**Figure 2:**
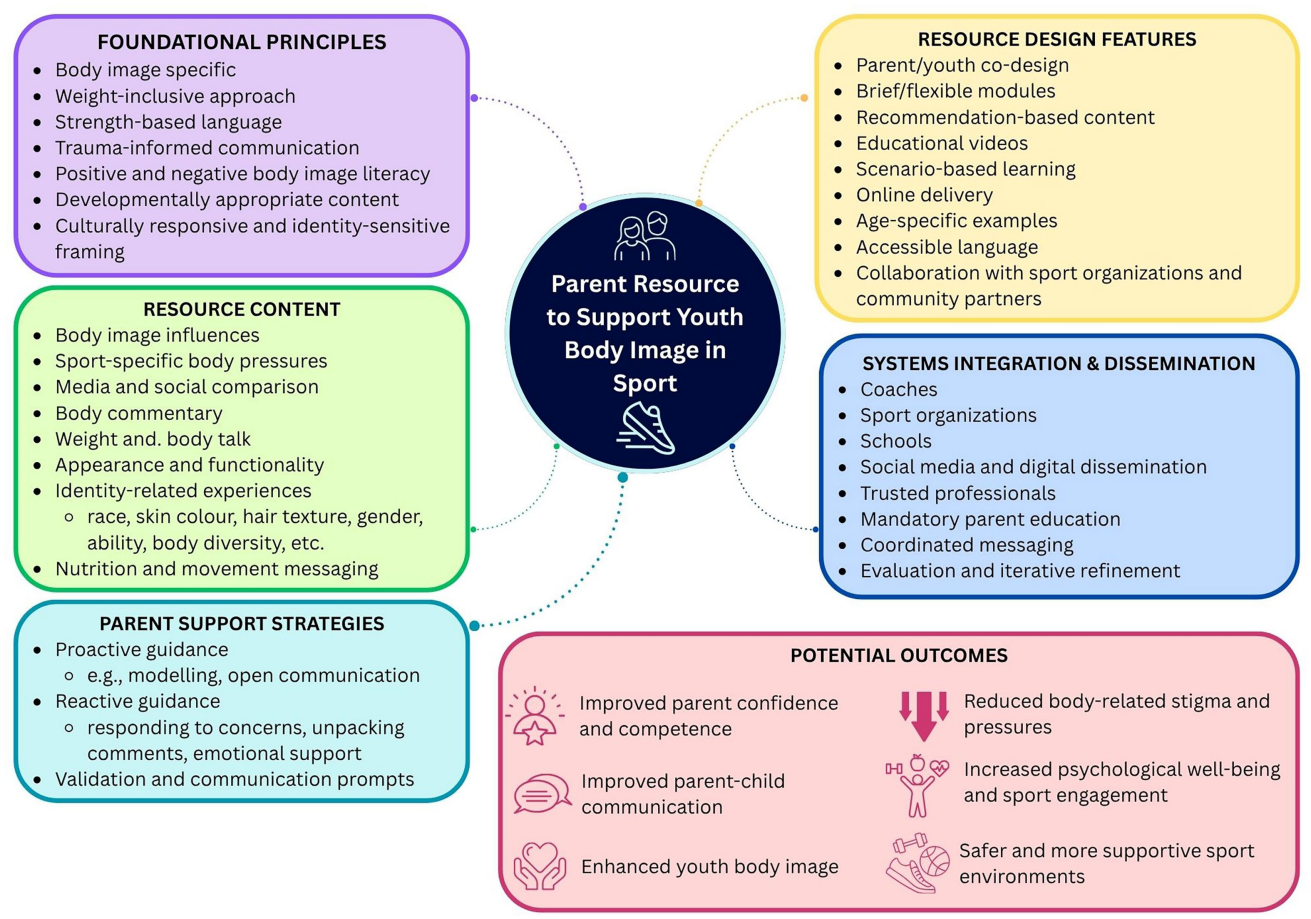
Integrative findings demonstrating the essential elements of future parent body image resources developed for youth sport.

The results of the scoping review and the parent consultation have been integrated into a figure that captures the essential elements of future parent body image resources developed for youth sport. The framework (see Figure 2) highlights five interconnected domains for resource development and delivery: foundational principles, resource content, parent support strategies, accessible resource design considerations, and systems integration and dissemination. Together, these domains emphasize that effective parent resources should move beyond individually focused psychoeducation approaches toward accessible, culturally responsive, developmentally appropriate, and sport-integrated supports that are embedded within the broader social environments influencing youth body image experiences. The framework also identifies potential outcomes associated with these approaches, including improved parent confidence and communication, enhanced youth body image, reduced body-related stigma, safer sport environments, and increased well-being and sport engagement.

## 4 Discussion

The present scoping review provides a comprehensive synthesis of the available published parent resources to support youth body image. This synthesis, in combination with the parent consultation, offers a broad overview of existing resources and identifies gaps that can inform the development of evidence-based parent-focused resources. Importantly, this review was intended to inform the future development of a sport-specific parent resource for youth body image by first synthesizing the broader landscape of existing parent-focused body image resources across contexts. A broader review scope was intentionally adopted given the limited availability of sport-specific parent body image resources identified in preliminary work.

Ten unique resources were identified that guide parents in supporting youth body image. The development of most resources was rigorous, wherein authors described using multiple evidence-informed methods. Despite rigorous development, parents in the focus group identified an inconsistency between how existing resources were designed and how parents prefer to engage with them, and this issue has been documented in previous parent-focused programs (Axford et al., 2012). Specifically, parents expressed a clear preference for recommendation-based resources and/or educational videos because they tend to favour brief, flexible, and easily accessible formats (Tongol et al., 2025). The disconnect between existing resource types and parent preferences highlights the importance of balancing evidence-based decision making with considerations of access, acceptability, and responsiveness to practical constraints (e.g., limited time, competing priorities). Existing resource formats may also reflect implicit assumptions regarding parental time, capacity, and health literacy. Parents in the consultation emphasized that lengthy or intensive programs were difficult to engage with, suggesting that many psychoeducational approaches may preferentially reach already-resourced families, while remaining less accessible to parents facing structural, financial, or time-related barriers (Koerting et al., 2013).

With respect to resource content, few resources primarily focused on body image. Instead, many addressed body image alongside weight, obesity, food/healthy eating practices, or self-esteem. Although many resources addressed these related topics, only body image-related content was extracted and analyzed in the present review. Nonetheless, the conflation of weight, eating behaviours, and body image may limit the relevance of several existing resources and could inadvertently reinforce weight-centric narratives that do not reflect the full range of children’s body image experiences. Moreover, embedding body image content within broader weight-, food-, or health-focused messaging may create interpretive ambiguity regarding whether resources are designed to support body image specifically or to promote weight-related behavioural outcomes. This lack of specificity may also perpetuate conceptual ambiguity within the field, where body image is often inconsistently defined and reduced to adjacent constructs despite being recognized as a unique multidimensional construct (Cash and Smolak, 2011; Sabiston et al., 2025). This pattern may reflect the continued dominance of weight-centric public health frameworks within parent-focused interventions, wherein body image is positioned as secondary to obesity prevention or health behaviour change. Such approaches risk reducing body image to an instrumental outcome of weight management rather than recognizing it as a multidimensional psychosocial construct worthy of direct support. Consistent with this concern, researchers indicate that weight-normative approaches exacerbate weight stigma, which is more strongly related to adolescent emotional distress than weight itself (Calogero et al., 2019; Hendy et al., 2025). Future resources targeting weight should therefore prioritize weight-inclusive approaches that minimize weight stigma; however, it is recommended that resources intended to support body image move beyond weight-centered framing and position body image as a distinct and central construct rather than a secondary outcome of weight or health promotion initiatives.

Furthermore, positive body image constructs were embedded into all resources, while negative body image appeared in about half of resources. Positive body image is a distinct construct that can co-occur with negative body image rather than reflecting an absence of negative body image (Tylka and Wood-Barcalow, 2015). The stronger emphasis on positive body image across resources may reflect a broader shift within intervention research toward strengths-based and preventative approaches; however, an exclusive focus on positivity may unintentionally underprepare parents to recognize distress, vulnerability, and early indicators of body image difficulties in youth. Helping parents to recognize and respond to children’s negative body image experiences is therefore also important (Rodgers et al., 2024) and future developed resources need to capture the duality and complexity of body image experiences.

Most existing resources included both proactive and reactive parenting strategies, aligning with parent accounts of desiring guidance on both how to initiate open-ended body image conversations and how to respond when children share body commentary they have received. To design useful and effective resources, researchers are encouraged to evaluate the relative effectiveness of recommendation strategies (i.e., proactive, reactive, or combined) and explore whether their importance varies by age or developmental stage of youth. Similar to broader psychoeducational intervention research (Cavaleri et al., 2011), many resources emphasized informational support and knowledge acquisition. However, parents in the consultation highlighted a stronger need for practical communication strategies, validation skills, and scenario-based guidance, suggesting that information alone may be insufficient to support meaningful relational change in parent–child body image interactions. In terms of social support, multiple types were represented across resources, reflecting the multifaceted role parents play in supporting their children. Researchers suggest that programs incorporating multiple types of social support are more effective than those offering fewer support types (Kuhn and Laird, 2014), highlighting a strength of existing resources. Notably, however, only half of resources included strategies aligned with validation support. Given evidence that parental emotion validation is related to greater adolescent disclosure of distressing experiences (Martin et al., 2018) and lower depressive symptoms (Ferrajão, 2020), parent resources would benefit from more explicitly incorporating validation-based recommendations. Further, considering social support types when designing parent resources, such as presenting recommendations rooted in emotional or validation support through scenario-based examples, may enhance usability.

### 4.1 Implications and future research

The results of the scoping review and parent consultation provide implications for resource development and inspire future research directions. Researchers are encouraged to evaluate the effectiveness of existing and new resources with parents and youth. Only one article in the present review examined both parent and youth outcomes, constraining a comprehensive understanding of how existing resources influenced parent behaviours and child outcomes. Further, a common limitation across the literature included in this review was the lack of evaluation with diverse identity groups, outlining an important area for future research. Researchers demonstrate that dominant body image approaches developed within White, Western contexts often fail to address culturally specific appearance concerns such as skin colour and hair texture (Capodilupo and Kim, 2014). Body image influences may impact diverse groups differently (Rodgers et al., 2024) which could impact the applicability of existing resources. Researchers are encouraged to develop new or revise existing resources to address diverse family experiences. In addition, parents in the focus group expressed surprise at the paucity of resources developed for parents of adolescents. Given that adolescence is characterized by body changes and a heightened attention on appearance and social standing (Markey, 2010), the development of parent resources tailored to adolescence would be valuable. The predominance of resources targeting parents of younger children may reflect prevention-oriented assumptions that body image concerns are most modifiable early in development (Hart et al., 2015). However, this emphasis may inadvertently overlook evidence that parents continue to shape body image experiences throughout adolescence, particularly through communication patterns, emotional support, and responses to sociocultural pressures (Rodgers and Chabrol, 2009). Finally, identifying the optimal dissemination strategy of resources is needed to improve reach. Parents in the focus group identified that using trusted sources (e.g., counselor, sport association) and integrating resources into existing systems (e.g., school, sport) could improve uptake. Therefore, a dissemination plan should be carefully designed within the resource development process.

When developing new parent resources, researchers are encouraged to use a co-design process (Sanders and Stappers, 2008), centering parents in the resource’s development by using collaborative approaches. Parents in the focus group reported being largely unaware of available body image resources, underscoring the importance of co-design approaches that enhance the relevance, acceptability, and usability of resources (Suna et al., 2025). Co-design processes could also intentionally consider social support types to ensure parents are equipped to provide effective support. An evaluation of whether resources improve parents’ delivery of different social support types and whether this translates to better child outcomes would be valuable. To ensure involvement of parents from diverse communities, strategies used to recruit parents and methods of participation need to be carefully considered. Intentional recruitment and participation strategies may improve the inclusivity and impact of future resource development efforts (Suna et al., 2025). In addition to parent involvement, including diverse youth in co-design may improve the relevance and effectiveness of resources by ensuring the content aligns with youths’ experiences. Finally, when available, best practice guidelines for resource development should be followed for the chosen resource type (e.g., AGREE II for guidelines; Brouwers et al., 2010).

Building on the need for co-designed, accessible, and well-disseminated parent resources, additional considerations are required when developing body image resources specific to youth sport contexts. While most existing resources discussed physical activity, they were not tailored to the sport environment. Informed by the parent consultation, parents expressed that those supporting youth in aesthetic sports may require additional guidance. However, previous research demonstrates that youth experience body-related pressures across both aesthetic and non-aesthetic sport (Kong and Harris, 2015; Lunde and Gattario, 2017). And so, parent resources would benefit from incorporating varied examples and scenarios that reflect diverse experiences across sport types and levels. Parents also emphasized the importance of coordinated efforts between parents, coaches, and sport organizations to foster positive sport environments, suggesting that aligning parent resources with coach education and organizational policies may promote more consistent and supportive body image messaging. Relatedly, parents discussed a greater likelihood of engaging with resources embedded into existing sport structures (e.g., mandatory league-based parent education). Similarly, existing resources largely frame body image support as an individual parenting responsibility, despite parents describing the broader adult environment (e.g., coaches, teachers, sport cultures) as highly influential. This tension suggests that parent-focused resources alone may be insufficient without parallel efforts targeting the wider sociocultural and sport systems in which youth body image develops. Involving potential dissemination partners, such as sport bodies and organizations, early in the resource development process may enhance their engagement as communication partners and improve the uptake and dissemination of parent body image resources (Elwy et al., 2022).

### 4.2 Strengths and limitations

This is the first scoping review to synthesize parent resources for youth body image. A wide range of resource types were included, which allowed for a broader understanding of the accessibility of resources for parents. In addition, the parent consultation strengthened the contribution of this scoping review by adding depth and contextualization to the review findings. As suggested by established scoping review methodological frameworks (Sabiston et al., 2022), consulting knowledge users improves the applicability and relevance of review findings. Within this present review, parent consultation enhanced the interpretation of the resources and ensured the results were aligned with parent perspectives.

Despite these strengths, there are limitations that require discussion. First, restricting the search strategy to English-language resources may have limited the review’s breadth and reduced the cultural relevance of the findings. Restricting the review to English-language resources may have disproportionately privileged Western conceptualizations of body image, parenting, and health communication. Body image experiences are shaped by culturally specific norms related to appearance, gender, race, skin colour, hair texture, food practices, and movement participation (Cash and Smolak, 2011) and therefore important culturally grounded resources may have been excluded. Similarly, peer-reviewed resources were synthesized, yet many parent-facing body image resources are likely developed and disseminated through community organizations, advocacy groups, healthcare systems, schools, sport organizations, and online platforms without accompanying academic publication. As a result, the present review may underrepresent practical and widely accessed non-academic resources currently available to parents. A review of these accessible resources (i.e., grey literature) would be valuable and informative. The analysis of the peer-reviewed resources was also constrained to the versions described in the articles when the original resources were no longer available or had been substantially updated since publication (e.g., website content that had changed; Diedrichs et al., 2016). Although it was a strength that most resources had been evaluated, heterogeneity in the measurement of body image constructs limited our ability to draw conclusions about effectiveness.

As an additional limitation, parents were not consulted prior to the search as recommended by the scoping review framework used (Sabiston et al., 2022). Although this may have limited the review’s scope, unpublished interviews with parents and consultations with experts and a health sciences librarian informed the development of the methods. Finally, all parents who participated in the consultation identified as women and were predominantly active, highly educated, and White. This lack of diversity limits the breadth of perspectives represented in our findings, which may reduce the applicability to parents with different identities, lived experiences, and structural realities. Further, the parents were already committed to supporting their children’s body image. Important perspectives from parents with limited time, capacity, or scheduling flexibility may not have been captured through the recruitment strategy used. The limited diversity of participating parents may also have shaped the types of concerns, dissemination preferences, and support strategies emphasized during consultation. Parents experiencing structural barriers, racialized appearance pressures, financial constraints, or limited access to organized sport may prioritize different resource needs, forms of support, and delivery approaches than those represented in the current sample.

## 5 Conclusion

Findings from this scoping review, combined with results from the parent consultation, highlight the need for evidence-informed, developmentally appropriate, culturally responsive parent resources for supporting youth body image in sport contexts. Future research should evaluate the effectiveness of resources on parent behaviours, youth outcomes, and across developmental stages and diverse identity groups. Engaging parents and youth through co-design, involving dissemination partners from the outset, and testing dissemination strategies within existing systems will be crucial to maximizing the impact of future parent resources.

## Data Availability

The datasets presented in this article are not readily available because the focus group transcript is not publicly available due to confidentiality agreements with participants and the sensitive nature of the qualitative data. Anonymized data can be made available upon reasonable request from the corresponding author, subject to ethical approval. Requests to access the datasets should be directed to catherine.sabiston@utoronto.ca.

## Footnotes

1
https://osf.io/9hq78
