# Supplementary material for: Body image resources for parents of youth: a scoping review
Source: Front Psychol. 2026 Jun 11;17:1869014. doi: 10.3389/fpsyg.2026.1869014 (PMC13293940; doi:10.3389/fpsyg.2026.1869014)
Supplement: Supplementary file 2 [file Table_2.docx]

Supplementary File 2

# Complete Search Strategy

**Medline via OVID**

| **Search line number** | **PCC component** | **Search term(s)** |
| --- | --- | --- |
| 1 | Population (youth) | exp parent/ |
| 2 | Population (youth) | (parent* or guardian* or caregiver* or carer* or mother* or father* or mom* or dad* or maternal or paternal or custodian*).tw,kf. |
| 3 | Concept (body image) | Body Image/ |
| 4 | Concept (body image) | Body image.tw,kf. |
| 5 | Concept (body image) | ((awareness or affect or appreciat* or assessment or attitude* or behav* or confiden* or critici* or cognition* or comment* or concern* or discrepancy or dissatisfaction or distortion or disturb* or drive for muscularity or embarrass* or esteem or envy or envious or experience? or function* or guilt or investment or perception? or pride or proud or project* or representation* or schema* or shame* or satisfaction or talk) adj2 (appearance or body)).tw,kf. |
| 6 | Concept (body image) | ((comment* or concern* or internalization* or embarrassment or emotion* or envy or envious or guilt or pride or shame or talk) adj2 weight).tw,kf. |
| 7 | Concept (body image) | (objectification or physical appearance or physical attractiveness or physique anxiety or thinness).tw,kf. |
| 8 | Concept (body image) | ((embarrassment or envy or shame or pride or guilt) adj2 (body-related or body-focused)).tw,kf. |
| 9 | Population | 1 or 2 |
| 10 | Concept | 3 or 4 or 5 or 6 or 7 or 8 |
| 11 | Population and concept | 9 and 10 |
| 12 | Limit by language and date | Limit 11 to (English language and yr=”2014-Current”) |

**EMBASE via OVID**

| **Search line number** | **PCC component** | **Search term(s)** |
| --- | --- | --- |
| 1 | Population (youth) | exp parent/ |
| 2 | Population (youth) | (parent* or guardian* or caregiver* or carer* or mother* or father* or mom* or dad* or maternal or paternal or custodian*).tw,kw. |
| 3 | Concept (body image) | Body Image/ |
| 4 | Concept (body image) | Body image.tw,kw. |
| 5 | Concept (body image) | ((awareness or affect or appreciat* or assessment or attitude* or behav* or confiden* or critici* or cognition* or comment* or concern* or discrepancy or dissatisfaction or distortion or disturb* or drive for muscularity or embarrass* or esteem or envy or envious or experience? or function* or guilt or investment or perception? or pride or proud or project* or representation* or schema* or shame* or satisfaction or talk) adj2 (appearance or body)).tw,kw. |
| 6 | Concept (body image) | ((comment* or concern* or internalization* or embarrassment or emotion* or envy or envious or guilt or pride or shame or talk) adj2 weight).tw,kw. |
| 7 | Concept (body image) | (objectification or physical appearance or physical attractiveness or physique anxiety or thinness).tw,kw. |
| 8 | Concept (body image) | ((embarrassment or envy or shame or pride or guilt) adj2 body-related or body-focused).tw,kw. |
| 9 | Population | 1 or 2 |
| 10 | Concept | 3 or 4 or 5 or 6 or 7 or 8 |
| 11 | Population and concept | 9 and 10 |
| 12 | Limit by language and date | Limit 11 to (English language and yr=”2014-Current”) |

**PsycINFO via OVID**

| **Search line number** | **PCC component** | **Search term(s)** |
| --- | --- | --- |
| 1 | Population (youth) | exp parents/ |
| 2 | Population (youth) | (parent* or guardian* or caregiver* or carer* or mother* or father* or mom* or dad* or maternal or paternal or custodian*).tw. |
| 3 | Concept (body image) | Body Image/ |
| 4 | Concept (body image) | Body image.tw. |
| 5 | Concept (body image) | ((awareness or affect or appreciat* or assessment or attitude* or behav* or confiden* or critici* or cognition* or comment* or concern* or discrepancy or dissatisfaction or distortion or disturb* or drive for muscularity or embarrass* or esteem or envy or envious or experience? or function* or guilt or investment or perception? or pride or proud or project* or representation* or schema* or shame* or satisfaction or talk) adj2 (appearance or body)).tw. |
| 6 | Concept (body image) | ((comment* or concern* or internalization* or embarrassment or emotion* or envy or envious or guilt or pride or shame or talk) adj2 weight).tw. |
| 7 | Concept (body image) | (objectification or physical appearance or physical attractiveness or physique anxiety or thinness).tw. |
| 8 | Concept (body image) | ((embarrassment or envy or shame or pride or guilt) adj2 body-related or body-focused).tw. |
| 9 | Population | 1 or 2 |
| 10 | Concept | 3 or 4 or 5 or 6 or 7 or 8 |
| 11 | Population and concept | 9 and 10 |
| 12 | Limit by language and date | Limit 11 to (English language and yr=”2014-Current”) |

**CINAHL via EBSCO**

| **Search line number** | **PCC component** | **Search term(s)** |
| --- | --- | --- |
| S1 | Population (youth) | (MH “Parents+”) |
| S2 | Population (youth) | TI (parent* or guardian* or caregiver* or carer* or mother* or father* or mom* or dad* or maternal or paternal or custodian*) OR AB (parent* or guardian* or caregiver* or carer* or mother* or father* or mom* or dad* or maternal or paternal or custodian*) |
| S3 | Concept (body image) | (MH “Body Image”) |
| S4 | Concept (body image) | TI ( (body image) ) OR AB ( (body image) ) |
| S5 | Concept (body image) | TI ( ((awareness or affect or appreciat* or assessment or attitude* or behav* or confiden* or critici* or cognition* or comment* or concern* or discrepancy or dissatisfaction or distortion or disturb* or drive for muscularity or embarrass* or esteem or envy or envious or experience? or function* or guilt or investment or perception? or pride or proud or project* or representation* or schema* or shame* or satisfaction or talk) N2 (appearance or body)) ) OR AB ( ((awareness or affect or appreciat* or assessment or attitude* or behav* or confiden* or critici* or cognition* or comment* or concern* or discrepancy or dissatisfaction or distortion or disturb* or drive for muscularity or embarrass* or esteem or envy or envious or experience? or function* or guilt or investment or perception? or pride or proud or project* or representation* or schema* or shame* or satisfaction or talk) N2 (appearance or body)) ) |
| S6 | Concept (body image) | TI ( ((comment* or concern* or internalization* or embarrassment or emotion* or envy or envious or guilt or pride or shame or talk) N2 weight) ) OR AB (((comment* or concern* or internalization* or embarrassment or emotion* or envy or envious or guilt or pride or shame or talk) N2 weight) ) |
| S7 | Concept (body image) | TI ( (objectification or physical appearance or physical attractiveness or physique anxiety or thinness) ) OR AB ( (objectification or physical appearance or physical attractiveness or physique anxiety or thinness) ) |
| S8 | Concept (body image) | TI ( ((embarrassment or envy or shame or pride or guilt) N2 body-related or body-focused) ) OR AB ( ((embarrassment or envy or shame or pride or guilt) N2 body-related or body-focused) ) |
| S9 | Population | S1 or S2 |
| S10 | Concept | S3 or S4 or S5 or S6 or S7 or S8 |
| S11 | Population and concept | S9 and S10 |
| S12 | Limit by language and date | Limit S11 to (English language and yr=”2014-Current”) |

**GenderStudies via EBSCO**

| **Search line number** | **PCC component** | **Search term(s)** |
| --- | --- | --- |
| S1 | Population (youth) | (DE “Parents”) |
| S2 | Population (youth) | TI (parent* or guardian* or caregiver* or carer* or mother* or father* or mom* or dad* or maternal or paternal or custodian*) OR AB (parent* or guardian* or caregiver* or carer* or mother* or father* or mom* or dad* or maternal or paternal or custodian*) |
| S3 | Concept (body image) | (DE “Body Image”) |
| S4 | Concept (body image) | TI ( (body image) ) OR AB ( (body image) ) |
| S5 | Concept (body image) | TI ( ((awareness or affect or appreciat* or assessment or attitude* or behav* or confiden* or critici* or cognition* or comment* or concern* or discrepancy or dissatisfaction or distortion or disturb* or drive for muscularity or embarrass* or esteem or envy or envious or experience? or function* or guilt or investment or perception? or pride or proud or project* or representation* or schema* or shame* or satisfaction or talk) N2 (appearance or body)) ) OR AB ( ((awareness or affect or appreciat* or assessment or attitude* or behav* or confiden* or critici* or cognition* or comment* or concern* or discrepancy or dissatisfaction or distortion or disturb* or drive for muscularity or embarrass* or esteem or envy or envious or experience? or function* or guilt or investment or perception? or pride or proud or project* or representation* or schema* or shame* or satisfaction or talk) N2 (appearance or body)) ) |
| S6 | Concept (body image) | TI ( ((comment* or concern* or internalization* or embarrassment or emotion* or envy or envious or guilt or pride or shame or talk) N2 weight) ) OR AB (((comment* or concern* or internalization* or embarrassment or emotion* or envy or envious or guilt or pride or shame or talk) N2 weight) ) |
| S7 | Concept (body image) | TI ( (objectification or physical appearance or physical attractiveness or physique anxiety or thinness) ) OR AB ( (objectification or physical appearance or physical attractiveness or physique anxiety or thinness) ) |
| S8 | Concept (body image) | TI ( ((embarrassment or envy or shame or pride or guilt) N2 body-related or body-focused) ) OR AB ( ((embarrassment or envy or shame or pride or guilt) N2 body-related or body-focused) ) |
| S9 | Population | S1 or S2 |
| S10 | Concept | S3 or S4 or S5 or S6 or S7 or S8 |
| S11 | Population and concept | S9 and S10 |
| S12 | Limit by language and date | Limit S11 to (English language and yr=”2014-Current”) |

**SPORTDiscus via EBSCO**

| **Search line number** | **PCC component** | **Search term(s)** |
| --- | --- | --- |
| S1 | Population (youth) | SU “Parents” |
| S2 | Population (youth) | TI (parent* or guardian* or caregiver* or carer* or mother* or father* or mom* or dad* or maternal or paternal or custodian*) OR AB (parent* or guardian* or caregiver* or carer* or mother* or father* or mom* or dad* or maternal or paternal or custodian*) |
| S3 | Concept (body image) | SU “Body Image” |
| S4 | Concept (body image) | TI ( (body image) ) OR AB ( (body image) ) |
| S5 | Concept (body image) | TI ( ((awareness or affect or appreciat* or assessment or attitude* or behav* or confiden* or critici* or cognition* or comment* or concern* or discrepancy or dissatisfaction or distortion or disturb* or drive for muscularity or embarrass* or esteem or envy or envious or experience? or function* or guilt or investment or perception? or pride or proud or project* or representation* or schema* or shame* or satisfaction or talk) N2 (appearance or body)) ) OR AB ( ((awareness or affect or appreciat* or assessment or attitude* or behav* or confiden* or critici* or cognition* or comment* or concern* or discrepancy or dissatisfaction or distortion or disturb* or drive for muscularity or embarrass* or esteem or envy or envious or experience? or function* or guilt or investment or perception? or pride or proud or project* or representation* or schema* or shame* or satisfaction or talk) N2 (appearance or body)) ) |
| S6 | Concept (body image) | TI ( ((comment* or concern* or internalization* or embarrassment or emotion* or envy or envious or guilt or pride or shame or talk) N2 weight) ) OR AB (((comment* or concern* or internalization* or embarrassment or emotion* or envy or envious or guilt or pride or shame or talk) N2 weight) ) |
| S7 | Concept (body image) | TI ( (objectification or physical appearance or physical attractiveness or physique anxiety or thinness) ) OR AB ( (objectification or physical appearance or physical attractiveness or physique anxiety or thinness) ) |
| S8 | Concept (body image) | TI ( ((embarrassment or envy or shame or pride or guilt) N2 body-related or body-focused) ) OR AB ( ((embarrassment or envy or shame or pride or guilt) N2 body-related or body-focused) ) |
| S9 | Population | S1 or S2 |
| S10 | Concept | S3 or S4 or S5 or S6 or S7 or S8 |
| S11 | Population and concept | S9 and S10 |
| S12 | Limit by language and date | Limit S11 to (English language and yr=”2014-Current”) |

**ERIC via ProQuest**

| **Search line number** | **PCC component** | **Search term(s)** |
| --- | --- | --- |
| S1 | Population (youth) | MAINSUBJECT.EXACT.EXPLODE("Parents") |
| S2 | Population (youth) | TI,AB,IF(parent* or guardian* or caregiver* or carer* or mother* or father* or mom* or dad* or maternal or paternal or custodian*) |
| S3 | Concept (body image) | TI,AB,IF(body image) |
| S4 | Concept (body image) | TI,AB,IF(body N/2 awareness) OR (appearance N/2 awareness) OR (body N/2 affect) OR (appearance N/2 affect) OR (body N/2 appreciat*) OR (appearance N/2 appreciat*) OR (body N/2 assessment) OR (appearance N/2 assessment) OR (body N/2 attitude*) OR (appearance N/2 attitude) OR (body N/2 behav*) or (appearance N/2 behav*) OR (body N/2 confiden*) OR (appearance N/2 confiden*) OR (body N/2 critici*) OR (appearance N/2 critici*) OR (body N/2 cognition*) OR (appearance N/2 cognition*) OR (body N/2 comment*) OR (appearance N/2 comment*) OR (body N/2 concern*) OR (appearance N/2 concern*) OR (body N/2 discrepancy) OR (appearance N/2 discrepancy) OR (body N/2 dissatisfaction) OR (appearance N/2 dissatisfaction) OR (body N/2 distortion) OR (appearance N/2 distortion) OR (body N/2 disturb*) OR (appearance N/2 disturb*) OR (body N/2 “drive for muscularity”) OR (appearance N/2 “drive for muscularity”) OR (body N/2 embarrass*) OR (appearance N/2 embarrass*) OR (body N/2 esteem) OR (appearance N/2 esteem) OR (body N/2 envy) OR (appearance N/2 envy) OR (body N/2 envious) OR (appearance N/2 envious) OR (body N/2 experience?) OR (appearance N/2 experience?) OR (body N/2 function*) OR (appearance N/2 function*) OR (body N/2 guilt) OR (appearance N/2 guilt) OR (body N/2 investment) OR (appearance N/2 investment) OR (body N/2 perception?) OR (appearance N/2 perception?) OR (body N/2 pride) OR (appearance N/2 pride) OR (body N/2 proud) OR (appearance N/2 proud) OR (body N/2 project*) OR (appearance N/2 project*) OR (body N/2 representation*) OR (appearance N/2 representation*) OR (body N/2 schema*) OR (appearance N/2 schema*) OR (body N/2 shame*) OR (appearance N/2 shame*) OR (body N/2 satisfaction) OR (appearance N/2 satisfaction) OR (body N/2 talk) OR (appearance N/2 talk) |
| S5 | Concept (body image) | TI,AB,IF(weight N/2 comment*) OR (weight N/2 concern*) OR (weight N/2 internalization*) OR (weight N/2 embarrassment) OR (weight N/2 emotion*) OR (weight N/2 envy) OR (weight N/2 envious) OR (weight N/2 guilt) OR (weight N/2 pride) OR (weight N/2 shame) OR (weight N/2 talk) |
| S6 | Concept (body image) | TI,AB,IF(objectification or “physical appearance” or “physical attractiveness” or “physique anxiety” or thinness) |
| S7 | Concept (body image) | TI,AB,IF(“body-related” N/2 embarrassment) OR (“body-focused” N/2 embarrassment) OR (“body-related” N/2 envy) OR (“body-focused” N/2 envy) OR (“body-related” N/2 shame) OR (“body-focused” N/2 shame) OR (“body-related” N/2 pride) OR (“body-focused” N/2 pride) OR (“body-related” N/2 guilt) OR (“body-focused” N/2 guilt) |
| S8 | Population | [s1] or [s2] |
| S9 | Concept | [s3] or [s4] or [s5] or [s6] or [s7] |
| S10 | Population and concept | [s8] and [s9] |
| S11 | Limit by language and date | Limit S10 to (English language and yr=”2014-Current”) |

**Sociological Abstracts via ProQuest**

| **Search line number** | **PCC component** | **Search term(s)** |
| --- | --- | --- |
| S1 | Population (youth) | MAINSUBJECT.EXACT("Parents") |
| S2 | Population (youth) | TI,AB,IF(parent* or guardian* or caregiver* or carer* or mother* or father* or mom* or dad* or maternal or paternal or custodian*) |
| S3 | Concept (body image) | MAINSUBJECT.EXACT("Body Image") |
| S4 | Concept (body image) | TI,AB,IF(body image) |
| S5 | Concept (body image) | TI,AB,IF(body N/2 awareness) OR (appearance N/2 awareness) OR (body N/2 affect) OR (appearance N/2 affect) OR (body N/2 appreciat*) OR (appearance N/2 appreciat*) OR (body N/2 assessment) OR (appearance N/2 assessment) OR (body N/2 attitude*) OR (appearance N/2 attitude) OR (body N/2 behav*) or (appearance N/2 behav*) OR (body N/2 confiden*) OR (appearance N/2 confiden*) OR (body N/2 critici*) OR (appearance N/2 critici*) OR (body N/2 cognition*) OR (appearance N/2 cognition*) OR (body N/2 comment*) OR (appearance N/2 comment*) OR (body N/2 concern*) OR (appearance N/2 concern*) OR (body N/2 discrepancy) OR (appearance N/2 discrepancy) OR (body N/2 dissatisfaction) OR (appearance N/2 dissatisfaction) OR (body N/2 distortion) OR (appearance N/2 distortion) OR (body N/2 disturb*) OR (appearance N/2 disturb*) OR (body N/2 “drive for muscularity”) OR (appearance N/2 “drive for muscularity”) OR (body N/2 embarrass*) OR (appearance N/2 embarrass*) OR (body N/2 esteem) OR (appearance N/2 esteem) OR (body N/2 envy) OR (appearance N/2 envy) OR (body N/2 envious) OR (appearance N/2 envious) OR (body N/2 experience?) OR (appearance N/2 experience?) OR (body N/2 function*) OR (appearance N/2 function*) OR (body N/2 guilt) OR (appearance N/2 guilt) OR (body N/2 investment) OR (appearance N/2 investment) OR (body N/2 perception?) OR (appearance N/2 perception?) OR (body N/2 pride) OR (appearance N/2 pride) OR (body N/2 proud) OR (appearance N/2 proud) OR (body N/2 project*) OR (appearance N/2 project*) OR (body N/2 representation*) OR (appearance N/2 representation*) OR (body N/2 schema*) OR (appearance N/2 schema*) OR (body N/2 shame*) OR (appearance N/2 shame*) OR (body N/2 satisfaction) OR (appearance N/2 satisfaction) OR (body N/2 talk) OR (appearance N/2 talk) |
| S6 | Concept (body image) | TI,AB,IF(weight N/2 comment*) OR (weight N/2 concern*) OR (weight N/2 internalization*) OR (weight N/2 embarrassment) OR (weight N/2 emotion*) OR (weight N/2 envy) OR (weight N/2 envious) OR (weight N/2 guilt) OR (weight N/2 pride) OR (weight N/2 shame) OR (weight N/2 talk) |
| S7 | Concept (body image) | TI,AB,IF(objectification or “physical appearance” or “physical attractiveness” or “physique anxiety” or thinness) |
| S8 | Concept (body image) | TI,AB,IF(“body-related” N/2 embarrassment) OR (“body-focused” N/2 embarrassment) OR (“body-related” N/2 envy) OR (“body-focused” N/2 envy) OR (“body-related” N/2 shame) OR (“body-focused” N/2 shame) OR (“body-related” N/2 pride) OR (“body-focused” N/2 pride) OR (“body-related” N/2 guilt) OR (“body-focused” N/2 guilt) |
| S9 | Population | [s1] or [s2] |
| S10 | Concept | [s3] or [s4] or [s5] or [s6] or [s7] or [s8] |
| S11 | Population and concept | [s9] and [s10] |
| S12 | Limit by language and date | Limit S11 to (English language and yr=”2014-Current”) |

**Social Work Abstracts via Ovid**

| **Search line number** | **PCC component** | **Search term(s)** |
| --- | --- | --- |
| 1 | Population (youth) | (Parents or parenting).sh. |
| 2 | Population (youth) | (parent* or guardian* or caregiver* or carer* or mother* or father* or mom* or dad* or maternal or paternal or custodian*).ti,ab. |
| 3 | Concept (body image) | Body Image.sh. |
| 4 | Concept (body image) | Body image.ti,ab. |
| 5 | Concept (body image) | ((awareness or affect or appreciat* or assessment or attitude* or behav* or confiden* or critici* or cognition* or comment* or concern* or discrepancy or dissatisfaction or distortion or disturb* or drive for muscularity or embarrass* or esteem or envy or envious or experience? or function* or guilt or investment or perception? or pride or proud or project* or representation* or schema* or shame* or satisfaction or talk) adj2 (appearance or body)).ti,ab. |
| 6 | Concept (body image) | ((comment* or concern* or internalization* or embarrassment or emotion* or envy or envious or guilt or pride or shame or talk) adj2 weight).ti,ab. |
| 7 | Concept (body image) | (objectification or physical appearance or physical attractiveness or physique anxiety or thinness).ti,ab. |
| 8 | Concept (body image) | ((embarrassment or envy or shame or pride or guilt) adj2 body-related or body-focused).ti,ab. |
| 9 | Population | 1 or 2 |
| 10 | Concept | 3 or 4 or 5 or 6 or 7 or 8 |
| 11 | Population and concept | 9 and 10 |
| 12 | Limit by language and date | Limit 11 to (English language and yr=”2014-Current”) |
