# Supplementary material for: Body image resources for parents of youth: a scoping review
Source: Front Psychol. 2026 Jun 11;17:1869014. doi: 10.3389/fpsyg.2026.1869014 (PMC13293940; doi:10.3389/fpsyg.2026.1869014)
Supplement: Supplementary file 3 [file Table_3.docx]

Supplementary File 3

# Chart form (Tables S1 and S2).

**Table S1. Study and Resource Characteristics and Resource Development.**

| **Authors (date)** | **Study design** | **Purpose** | **Resource Type** | **Resource Format** | **Methods for Developing Resource** | **Target Age of Resource** |
| --- | --- | --- | --- | --- | --- | --- |
| Baber, Gillison, & Grey (2023) | Qualitative | Explore the perceived relevance, utility and acceptability of new guidance for parents on talking to children about weight | Guidelines | Online text | Systematic review  Engagement of key groups (parents, children, public health practitioners)  Delphi study  Interviews with parents Interviews with experts  Acceptability study | 4-11 years |
| Carbonneau, Hamilton, & Musher-Eizenman (2021) | Review | Provide an overview of research on parental practices related to body  image, strategies for creating a positive family climate around  food, and food parenting practices | Recommendations | Online text | Literature review | N/A |
| Diedrichs, Atkinson, Garbett, Williamson, Halliwell, Rumsey, Leckie, Sibley, & Barlow (2016) | Quantitative | Evaluate the impact of a low-intensity  internet-based body image intervention for parents  among mother-daughter dyads | Website | Online text | Engagement of key groups (researchers, psychologists, youth workers, professional writers) | 11-14 years |
| Eldridge, Paul, Bailey, Ashe, Martz, & Lynch (2016) | Quantitative | Evaluate the efficacy of a parent-only face-to-face (experimental) healthy lifestyles intervention to improve child body image | Program | Online video  Print  Workshops/sessions | Focus groups with parents  Review (e.g., scoping, literature)  Pilot test with parents  Feasibility study | 8-12 years |
| Forbes, Paxton, & Yager (2024) | Quantitative | Evaluate the efficacy and acceptability of Raising Confident Girls, a face-face, interactive, etiologically based, and multi-session intervention when delivered to a large group of mothers within the school context | Program | Online video  Workshops/Sessions | Review (literature)  Adapted previously tested programs  Informed by conversations with parents  Prior research and experience of authors  Acceptability study | Year 8 students (approximately 13 years) |
| Gillison, Grey, Baber, Chater, Atkinson, & Gahagan (2023) | Mixed methods | Develop guidance for parents on constructive ways to talk to children of primary school age about weight | Guidelines | Online text | Systematic reviews  Interviews with children  Delphi study | 4-11 years |
| Hart, Damiano, Chittleborough, Paxton, & Jorm (2014) | Mixed methods | Develop guidelines for parents to prevent body dissatisfaction and unhealthy eating patterns in preschool children, by using the Delphi method for establishing expert consensus | Guidelines | Online text | Delphi study  Systematic search and content analysis of publications on parenting strategies addressing the development of body image and healthy eating patterns in children | 2-6 years |
| Hart, Damiano, & Paxton (2016) | Quantitative | Evaluate Confident Body, Confident Child, an intervention for parents of 2- to 6-year-old children, designed to promote body satisfaction, healthy eating, and weight management in early childhood. | Program | Online text  Print  Workshops/Sessions | Review (literature)  Delphi study  Focus groups with parents  Interviews with experts  Pilot test with parents (workshops to get feedback on design, language, and content of prototype with survey/verbal feedback) | 2-6 years |
| Hart, Damiano, Li-Wai-Suen, & Paxton (2018) | Quantitative | Evaluate Confident Body, Confident Child, a universal parenting resource designed to promote positive body image and healthy eating patterns in children aged 2–6 years, at 6- and 12-months follow-up. | Program | Online text  Print  Workshops/Sessions | Review (systematic)  Delphi study  Survey with parents  Previous research and behaviour change theories | 2-6 years |
| Hill, Hart, & Paxton (2020) | Quantitative | Conduct an exploratory follow-up study assessing whether there were significant differences among children of the parents involved in the original Confident Body, Confident Child parent trial on measures of eating patterns, child body image and weight bias | Program | Online text  Print  Workshops/Sessions | Review (systematic)  Focus groups with parents  Interviews with experts  Delphi study (see Hart et al. 2014, 2016)  Previous research and behaviour change theories | 2-6 years |
| Horton (2024) | Qualitative | Explore the experiences of caregivers who integrate the concept of body neutrality in their parenting | Co-created theory | Online text | Interviews with parents | Under 18 years |
| Klupt, Oreskovich, Bernard-Genest, Patel, Chu, Dettmer, Walsh, Strom, McPherson, Strub, Steinberg, Steineggar, & Hamilton (2020) | Quantitative | Create an educational video, and to assess the effect on parents' self-efficacy for communicating about weight-related issues with their children | Educational video | Online video | Review (scoping)  Interviews with parents  Survey of experts  Pilot test with parents (non-randomized, prospective, single arm)  Acceptability study | N/A |
| McCabe, Mellor, & Mealey (2016) | Quantitative | Investigate whether an educational programme for parents focusing on body image issues of boys aged 3-6 years is beneficial | Program | Print  Workshops/Sessions | No information on development | 3-6 years |
| Meskin, Colvin, & Hart (2021) | Quantitative | Pilot the first research trial of the Confident Body, Confident Child program in the United States | Program | Online text  Print  Workshops/Sessions | Literature review  Delphi study  Focus groups with parents | 2-6 years |
| Twomey & Gillison (2025) | Qualitative | To explore the acceptability and usability of parent guidance for talking to children about their weight from the perspective of general practitioners | Guidelines | Online text | Evidence review  Delphi study  Engagement of key groups (parents, children, public health practitioners. Academics, health care providers) | 4-11 years |

**Note.** In this review, guidelines were defined as evidence-informed documents that provide structured and formal instructions to direct actions on a specific topic, recommendations were defined as a set of specific suggestions or proposed actions on a topic, programs were defined as an organized set of activities designed to achieve defined outcomes or a certain population. Other resource types did not fit within these options and were labelled using the author’s terminology.

**Table S2. Resource Content and Evaluation**

| **Authors (date)** | **Topics** | **Identity factors in resource** | **Evaluation Design (if applicable)** | **Population and Sample Size** | **Measures** | **Main Findings** |
| --- | --- | --- | --- | --- | --- | --- |
| Baber, Gillison, & Grey (2023) | Body image influences  Positive body image  Negative body image  Identity factors  Health behaviours | Weight | Acceptability study | Parents (*n* = 12)  Primary health providers (*n* = 15) | Individual interview schedule | Guidance was perceived as important, needed, and helpful.  Normalized weight conversations, reduced feelings of blame or guilt, increased confidence, and empowered parents to have weight conversations.  Some felt the guide was too general and wanted more applied suggestions.  Language and content were realistic. |
| Carbonneau, Hamilton, & Musher-Eizenman (2021) | Body image influences  Positive body image  Health behaviours | N/A |  |  |  |  |
| Diedrichs, Atkinson, Garbett, Williamson, Halliwell, Rumsey, Leckie, Sibley, & Barlow (2016) | Body image influences  Positive body image  Identity factors | Gender | Randomized controlled trial | Daughter-mother dyads (*n* = 235 dyads) | The Appearance Esteem  and Weight Esteem subscales of the Body Esteem Scale for Adults and Adolescents (Mendelson et al., 1997)  General Internalization and Pressures subscales of the Sociocultural  Attitudes Toward Appearance Scale-3 (Thompson et al., 2004)  Perceived Sociocultural Pressures Scale (Stice & Bearman, 2001)  Social Comparison to Models and Peers Scale  (Jones, 2001)  Teasing items validated in Project Eat-III (Neumark-Sztainer et al., 2007)  Appearance Conversations with Friends subscale of Culture Among Friends (Jones et al., 2004)  Single-item (“How  often do you speak to your mother/daughter about body image?”)  Maternal Pressures Scale (Corning et al.,  2010)  Rosenberg Self-Esteem Scale (Rosenberg,  1965)  Positive and Negative Affect Schedule (Watson, Clark, & Tellegen, 1988), with a child version used for  daughters (5 items; Ebesutani et al., 2012)  Purpose-built questions | Relative to control, mothers who viewed the website reported higher self-esteem, weight esteem, lower negative affect, engaged in more self-reported conversations with their daughters about body image, and were more likely to report seeking additional support for body image issues.  Daughters whose mothers viewed the website had higher self-esteem and reduced negative affect. There were no differences on daughters’ body image. |
| Eldridge, Paul, Bailey, Ashe, Martz, & Lynch (2016) | Body image influences  Positive body image  Identity factors  Health behaviours | Gender  Age | Intervention |  | Anthropometric measures (height, weight, blood pressure, resting heart rate)  Youth Risk Behaviour Survey (Centers for Disease Control and Prevention, 2009)  Children's Body Image Scale (Truby & Paxton, 2002)  The Body Esteem Scale (Mendelson et al., 1996)  Sociocultural Attitudes Towards Appearance Questionnaire (Heinberg et al., 1995) | Lower BMIz scores and overweight dissatisfaction and higher body esteem and appearance attitudes. No effect on body size perception. |
| Forbes, Paxton, & Yager (2024) | Body image influences  Positive body image  Negative body image  Identity factors  Health behaviours | Gender  Age | Quasi-experimental acceptability and effiacy | Mothers (*n* = 120) | Purpose built questions measuring Parenting Knowledge and Parent Skills & Confidence  Body Appreciation Scale (Avalos et al., 2005)  Body Esteem Scale for Adolescents and Adults (Mendelson et al., 2001)  Ideal-Body Stereotype Scale - Revised (Stice et al., 1996)  Physical Appearance Comparison Scale (Thompson et al., 1991)  Appearance Conversation Scale (Jones et al., 2004)  Rosenberg Self-esteem Scale shortened (Neumark-Sztainer et al., 2007; Rosenberg, 1965)  Dutch Eating Behaviour Questionnaire (van Strien et al., 1986)  Maternal Pressure Scale (Corning et al., 2010)  Role Modelling of Body Image Questionnaire (Damiano et al., 2019)  Purpose built measure used previously (Diedrichs et al., 2010) to measure program acceptability  Program attendance | Mothers who participated in the program had higher body esteem and body appreciation compared to the comparison group. Participation also improved mother’s knowledge, confidence, and skills for parenting, and improved role modeling of positive body image. Low attrition rates and high program ratings. |
| Gillison, Grey, Baber, Chater, Atkinson, & Gahagan (2023) | Body image influences  Positive body image  Negative body image  Identity factors  Health behaviours | Weight |  |  |  |  |
| Hart, Damiano, Chittleborough, Paxton, & Jorm (2014) | Body image influences  Positive body image  Negative body image  Identity factors  Health behaviours | Gender  Puberty  Culture |  |  |  |  |
| Hart, Damiano, & Paxton (2016) | Body image influences  Positive body image  Negative body image  Identity factors  Health behaviours | Gender  Weight  Age | Randomized controlled trial | Parents (*n* = 345) | Knowledge Test for Body Image and Eating Patterns in Childhood (Damiano et al., 2015)  Parenting Intentions for Body Image and Eating Patterns in Childhood (Damiano et al., 2015)  Four measures of family mealtimes (Fulkerson et al., 2006)  Six parental feeding practice scales (Rodgers et al., 2013) | Lower parent intentions to use behaviors that increase the risk of negative body attitudes or unhealthy eating in their children, feeding practices associated with childhood overweight, and television watching during family meals. Higher parents’ intentions to use positive behaviors and knowledge of child body image and healthy eating patterns. |
| Hart, Damiano, Li-Wai-Suen, & Paxton (2018) | Body image influences  Positive body image  Negative body image  Identity factors  Health behaviours | Gender  Weight  Age | Randomized controlled trial | Parents (*n* = 345) | Knowledge Test for Body Image and Eating Patterns in Childhood (Damiano et al., 2015)  Parenting Intentions for Body Image and Eating Patterns in Childhood (Damiano et al., 2015)  Four measures of family mealtimes (Fulkerson et al., 2006)  Six parental feeding practice scales (Rodgers et al., 2013) | Higher knowledge, parenting intentions, and lower parental feeding practice of weight restriction. No other significant differences between groups. |
| Hill, Hart, & Paxton (2020) | Body image influences  Positive body image  Negative body image  Identity factors  Health behaviours | Gender  Weight  Age | Interviews | Children (*n* = 89) | Dutch Eating Behavior Questionnaire (van Strien & Oosterveld, 2008)  Body Esteem Scale (Mendelson & White, 1982)  Figure rating scale (Tiggemann & Pennington, 1990)  Body mass index | Children of parents who engaged in the program reported higher body esteem and lower external eating. Control group reported higher weight bias. |
| Horton (2024) | Body image influences  Positive body image  Negative body image  Body neutrality  Identity factors  Health behaviours | Weight |  |  |  |  |
| Klupt, Oreskovich, Bernard-Genest, Patel, Chu, Dettmer, Walsh, Strom, McPherson, Strub, Steinberg, Steineggar, & Hamilton (2020) | Body image influences  Positive body image  Identity factors  Health behaviours | Weight  Gender | Non-randomized pilot trial | Parents (*n* = 57) | Purpose built self-efficacy questions guided by Bandura (2006)  Purpose built questions on satisfaction and relevance adapted from Whittemore et al. (2010) | Higher self-efficacy in “raising the issue of weight” and “answering questions or concerns”. Parents with concerns about their child being overweight had lower perceived self-efficacy scores compared to parents with no concerns about their child’s weight. Video was positively received and of relevance to parents. |
| McCabe, Mellor, & Mealey (2016) | Body image influences  Positive body image  Negative body image  Identity factors  Health behaviours | Gender  Weight | Randomized controlled trial | Parents (*n* = 118) | Purpose built questionnaire on the level of perceived knowledge on body image of preschool boys and role of parents in son’s body image development  Body Appreciation Scale (Avalos et al., 2005) | Parents in the intervention group reported higher perceived knowledge and body appreciation compared to control group |
| Meskin, Colvin, & Hart (2021) | Body image influences  Positive body image  Negative body image  Identity factors  Health behaviours | Gender  Weight  Age | Pilot pre-test post-test | Parents (*n* = 92) | Knowledge Test for Body Image and Eating Patterns in Childhood (Damiano et al., 2015)  Weight Bias Internalization Scale–Modified Version (Pearl & Puhl, 2014)  State Body Appreciation Scale–2 (Homan, 2016) | Parents reported higher knowledge post-test. No significant differences were found in weight bias internalization and body appreciation. |
| Twomey & Gillison (2025) | Body image influences  Positive body image  Negative body image  Identity factors  Health behaviours | Weight | Interviews | Practicing general practitioners (*n* = 15) | Individual interview guide | Guidance was perceived as acceptable, relevant, and useful for having conversations with parents about their child’s weight.  Preferred a printed version that could be handed to parents.  Language and content were appreciated; however, the length was a concern for some. |
